# Nursing home staff experiences with providing dementia care

**DOI:** 10.1002/alz.083711

**Published:** 2025-01-09

**Authors:** Carin M. Wong, Dominique H. Como, Cara A. Lekovitch, Felicia M Chew, Cindy Kuziak, Natalie E Leland

**Affiliations:** ^1^ University of Pittsburgh, Pittsburgh, PA USA; ^2^ Thomas Jefferson University, Philadelphia, PA USA; ^3^ Study Advisory Committee Member, Pittsburgh, PA USA

## Abstract

**Background:**

Caring for nursing home residents with dementia can be challenging. Staff who work in nursing homes tend to have high staff turnover. In order to help with staff retention, there needs to be an understanding of the job role. There is limited research examining staff experiences providing nursing home dementia care. Prior research has largely focused on the negative experiences of staff, such as difficulty managing residents’ behaviors, staff burnout, and staff stress, instead of positive experiences. It is important to understand the collective staff experience (i.e., both positive and negative experiences) in delivering dementia care, which can inform the development of job descriptions and hiring of nursing home staff who are a good fit.

**Methods:**

This qualitative study was embedded within a pragmatic clinical trial. Participants were interviewed to gather staff perspectives on two approaches to dementia care training (team‐ and problem‐based). Interviews were conducted with 327 staff members representing a range of job roles. A rapid qualitative analysis approach was used to analyze the interviews. During the analysis, staff experiences in providing dementia care were consistently described. A thematic analysis approach was used to further analyze the data describing positive and negative experiences providing dementia care.

**Results:**

Staff described experiencing both positives and negatives caring for residents with dementia. Positive experiences included three themes: the inter‐personal relationship with residents (e.g., learning about residents’ likes, dislikes, history), the emotional experiences from providing care (e.g., having a sense of purpose), and the cognitive demands of the job (e.g., using different strategies to distract residents). Challenges included two themes: the physical and cognitive demands of the job (e.g., not being able to manage residents’ behaviors) and the emotional impact on staff (e.g., affecting staff mental health).

**Conclusions:**

Nursing home staff report positive and negative experiences while providing care to people with dementia. In order to ensure appropriate staff are being hired, nursing home administrators and hiring personnel should highlight the positives and describe the potential negative experiences that staff may have in caring for residents with dementia.

